# On the Extermination of the Small-Pox, in Reply to a Paper in the Last Number of the Medical and Physical Journal, on That Subject

**Published:** 1803-05-01

**Authors:** 


*On the* EXTERMINATION *of the* SMALL-POX, *in reply to a Paper in the last Number of the Medical and Physical Journal, on that Subject.*

MANY have advanced objections against extending the practice of Vaccine Inoculation, because they deemed it insufficient to secure the constitution from variolous contagion, or because they imagined that by sowing the seeds of future maladies, it might prove eventually more destructive to the human race than the Small-pox itself. The practice has lately been condemned on a very different ground, for no other reason but because the vaccine is an effectual security against the horrid scourge which has so long depopulated the species, and is likely to effect its extermination from the face of the globe. The argument may be exhibited clearly in the following manner. The Small-pox is a most destructive disease, therefore no attempts should be made to prevent its ravages. The Cow-pox will secure the constitution from variolous contagion, therefore medical men are blamable in recommending its adoption.

These strange conclusions the writer endeavours to support and to justify in a serious tone.

It appeared on a superficial perusal of his paper, that he was treating the subject in a style of irony; that he professed to espouse and recommend opinions, which, by the weak support he gave them, he meant in reality to ridicule or disprove; but on farther examination it was conceived, that he was truly serious in asserting that the Small-pox ought not to be prevented, because it occasions so great a mortality among mankind. It will, however, be proper to state the intermediate steps of the argument by which he is brought to this remarkable conclusion.

1. As great a number of men exist at all times as the earth is able to support.

2. Vice and misery were understood to prevent an increase of population.

3. Disease is a species of misery, and Small-pox is a species of disease; therefore,

The Small-pox ought not to be prevented, for some other malady, equally or even more destructive, would probably be substituted in its stead.

As to the first position, it is very questionable if not absolutely false. Islands thinly peopled, or entirely uninhabited, wide tracts of uncultivated land in different parts of the globe, are sufficient to remove the apprehension that the earth cannot support a greater number of inhabitants than at present exist. Cultivation alone is wanting. The earth would be more bountiful if men were more industrious.

> Labor omnia vincit
> Improbus.

The second position, which asserts that vice and misery are introduced, in order to prevent an unsupportable population, requires proof. The origin or final cause of vice and misery is a subject which baffles our mental powers, and defies explanation. It is a depth which reason cannot fathom. Fertility of invention and skilfulness of discussion, the accuracy of logic, and the dextrous subtlety of metaphysics, have been exercised to no purpose.

purpose. The greater part of mankind still confess their doubts and dissatisfaction on this point; and the small number who imagine they can solve the difficulty, and propose various theories with this view, have only served to convince others more strongly of the impenetrable darkness which envelops the subject they attempt to illustrate and explain.

But the writer of the paper to which I reply, seems to consider the origin of vice and misery as a problem very easy to be demonstrated; and asserts, with the utmost confidence, without taking the trouble to advance one proof in support of his position, that the final cause of vice and misery is to prevent a greater population than the earth will support.

A weak and precipitate solution of a difficult and perhaps unanswerable enquiry, ποθεν το κακον.

The efforts which have been made to explain this subject, and the doubts which are still entertained, evidently shew, that the writer's second position requires proof before it can be admitted as true, and allow great room to suppose, that if he had attempted to elucidate the point, he would, like others, have failed. It is matter of surprize, that an assertion of this nature should be chosen as the principal support of an opinion, so important that, if received, it would destroy the beauty, the vigor, and the lives of millions.

Against the third stage of the argument I do not contend. Disease is, without doubt, a species of misery, and the Small-pox is a species of disease.

But the conclusion cannot be allowed, for it is deduced from premises which are doubtful or false.

Besides, the conclusion, if it has any weight at all, has too much. It proves the impropriety of attempting to prevent any other disease as well as the Small-pox. It is absurd to imagine that it applies to one and not to all. Can any reason be given why we should endeavour to prevent a disease which is mild, and not one which is highly destructive. There are many maladies which afflict us, and contribute to thin the ranks of mankind. According to the author's mode of reasoning, it is improper to adopt measures for the removal of any disease, lest population should increase too rapidly for support.

All complaints, whether mild or violent, or innocent or destructive, from the slightest indisposition to the most formidable malady, must be borne patiently, without the smallest effort to alleviate, remove, or prevent them.

The Faculty are only counteracting the intentions and designs of Providence, when they endeavour to preserve the health of their fellow-creatures. The physician who prescribes, and the patient who uses the medicine, are both equally culpable. These absurd consequences, evidently deducible from the author's conclusion, will, it is hoped, induce him to abandon the strange opinion which he has attempted to support. Far from adopting his sentiments, I cannot forbear thinking, that as it is one of the grand principles of our nature to wish to avoid pain and death, so it is not improper, either in us to prevent disease, or in the Faculty to warn and instruct us to prevent it; that if we are able to destroy a malady which, in every age, devours its millions of victims, it becomes a duty to do it; that if the author of Nature wished to diminish, or stop the increase of population, by the continuance of the Small-pox, he would not have permitted the means of preventing this dreadful disease to be discovered; that since he has permitted the means to be discovered, he commands us, in our instinctive principles, as we regard our own happiness, the happiness of our children, our relatives or friends, our neighbours, our countrymen, and all mankind, to embrace with eagerness and gratitude the blessing which is offered to our acceptance. Even if we argue on the author's principle, and imbibe his apprehensions on the subject of an increased population, we might, with earnestness, recommend and adopt the practice of Vaccine Inoculation. During the late ravages of war, such multitudes have fallen in the field of battle, that it seems necessary, in order to replenish the world, that some destructive disease should be annihilated.

I do not think, therefore, that medical gentlemen are unjustifiable, but, on the contrary, highly deserving of applause, for the steps which they have taken to effect the extermination of the Small-pox.

And I must say, in conclusion, that it has been, is, and ever will be, my custom, when I am attacked with any complaint, instead of letting it take its course, lest population should increase too rapidly, to seek and employ the most likely means of alleviating or removing it. I have often yielded to the solicitations of my friends, who advised me to all such precautions as might prevent cold, or rheumatism, or fever, or any malady to which, on different occasions, I might be exposed; and pursuing the same plan of conduct, I have taken particular care to secure cure the constitution of my children from the attacks of the Small-pox, by having them inoculated as early as possible with the Vaccine.

